# Two Genealogies of Human Values: Nietzsche Versus Edward O. Wilson on the Consilience of Philosophy, Science and Technology

**DOI:** 10.1007/s11948-019-00095-2

**Published:** 2019-02-26

**Authors:** Charles C. Verharen

**Affiliations:** Department of Philosophy, Howard University, Washington, DC 20059 USA

**Keywords:** Nietzsche, Edward O. Wilson, Philosophy, Science, Technology, Human values

## Abstract

In the twenty-first century, Stephen Hawking proclaimed the death of philosophy. Only science can address philosophy’s perennial questions about human values. The essay first examines Nietzsche’s nineteenth century view to the contrary that philosophy alone can create values. A critique of Nietzsche’s contention that philosophy rather than science is competent to judge values follows. The essay then analyzes Edward O. Wilson’s claim that his scientific research provides empirically-based answers to philosophy’s questions about human values. Wilson’s bold new hypothesis about the ‘social conquest of the earth’ challenges Nietzsche’s vision of philosophy’s mission. Confronting both Nietzsche and Wilson, the essay then considers three theoretical proposals for a consilience of philosophy, science, engineering and technology. The conclusion presents a working African model of consilience that addresses the existential problem of poverty in the Global South.

## Introduction

I am still waiting for a philosophical *physician*, in the exceptional sense of the word—one who has to pursue the problem of the total health of a people, time, race or of humanity—to master the courage to push my suspicion to its limits and to risk the proposition: what was at stake in all philosophizing hitherto was not at all ‘truth’ but something else—let us say health, growth, future, power, life (Nietzsche’s emphasis, 2001/1887, 2nd Edition Preface [2]: p. 6).^1^

In the twenty-first century, prominent scientist Stephen Hawking (Hawking and Mlodinow 2010: p. 5) announced that “philosophy is dead.” Stephen Pinker (2011, 2013, 2018) has claimed that virtually all the truths professed by religion are false. In both cases, the inference is that answers to perennial questions about human values^2^ are to be discovered through the empirical sciences. Science alone is capable of answering our most pressing questions about where we come from and how we should live.

Twenty-first century philosophy confronts at least two severe challenges. The lesser comes from scientists like Hawking, Richard Dawkins, and Neil deGrasse Tyson who claim that philosophy is dead. A prescient Friedrich Nietzsche (2001/1887, V [373]: p. 239) endows such scientists with the title “Mr. Mechanic, who nowadays likes to pass as a philosopher….” The greater challenge comes from scientists who announce the sixth mass extinction of life on earth (Gardiner 2011).

Over 50 years ago, Snow (1982/1959: p. 98) warned about the gulf between the sciences and humanities: “It is dangerous to have two cultures which can’t or don’t communicate. In a time when science is determining much of our destiny, that is, whether we live or die, it is dangerous in the most practical sense.” Snow’s great fear was the destruction of the earth’s food chain through nuclear weapons of mass destruction (Schell 2000). In what some now call the Anthropocene Age, global climate change threatens the earth with the sixth mass extinction (Kolbert 2014). The growing cleavage between the sciences and humanities forestalls communication that can address this peril.

In the nineteenth century, Nietzsche carves out a unique role for philosophers as “guarantors of the future” (2014/1887, III [14]: p. 91; see Stegmaier 2016). Philosophy’s method is to create new values, rank-order values, and furnish genealogies of values. Science, he claims, cannot perform that task: “science itself never creates values” (ibid. III [25]: p. 113). Against Nietzsche, Edward O. Wilson in two of his books, *Consilience: The Unity of Knowledge* (1998) and the more recent *The Meaning of Human Existence* (2014) proposes to discover the meaning of human existence through research in the biological sciences. He appears to offer a “consilience” or the “jumping together” of the sciences and humanities. While William Whewell (1840) introduced the term to refer to the consilience of inductions taking place from different classes of facts, Wilson defines *consilience* as a method of combining the methodologies of science and the humanities to create new knowledge. However, Wilson’s vision of such a consilience is “to [turn] as much philosophy as possible into science” (1998: pp. 11–12).

Wilson (ibid: p. 96) claims that philosophy has failed in its mission of assessing values because of its support for “failed models of the brain.” Wilson believes that “a few philosophers” will resist his proposal for uniting the sciences and humanities. They will charge him with poaching on their own territory with accusations of “conflation, simplism, ontological reductionism, scientism….” In the end (ibid: pp. 11–12), he declares that philosophy “as the contemplation of the unknown is a shrinking dominion,” scheduled for replacement by science.

The paper begins with an examination of Nietzsche’s argument that only philosophy is competent to conduct the revaluation of all values by destroying old values that no longer encourage the growth of life, thereby ensuring its continuance. A critique of Nietzsche’s conviction that only philosophy can create and judge values follows. Then comes an analysis of Wilson’s contention that philosophy can no longer exercise its mission of evaluation because it has lost contact with the sciences: philosophy to the degree possible must become a science.

Finally, this essay explores three solutions to the stand-off between Nietzsche and Wilson. Each solution proposes a true “consilience” of philosophy and science. The conclusion offers a practical model of consilience of philosophy and the STEM disciplines that addresses the existential problem of poverty in the Global South.

## Nietzsche’s Consilience of Philosophy and Science

Nietzsche (1995/1872–74, 19 [62]: p. 23) makes the bold claim that philosophy is both a science and an art and so difficult to categorize: “Great quandary: whether philosophy is an art or a science. In its aims and in its results it is an art. But its means, conceptual representation, it shares with science. It is a form of poetic artistry. It cannot be categorized: consequently we must invent and characterize a species for it.”

Bernard Williams (2001: p. x) in his introduction to the Cambridge translation of the *Gay Science* points out that Nietzsche’s *Wissenschaft,*

unlike the English word “science” in its modern use, does not mean simply the natural and biological sciences – they are more specifically, *Naturwissenschaft*. It means any organized study or body of knowledge, including history, philology, criticism and generally what we call the “humanities,” and that is often what Nietzsche has in mind when he uses the word in the text (it is often translated as “science,” for want of a brief alternative).

In what follows, the context of the citations clarifies Nietzsche’s differentiation of the terms *philosophy* and *science*.

While Nietzsche envisions the consilience of philosophy and science, he insists that only philosophy can create values. He goes so far as to claim that philosophers are the “guarantors of the future” (2014/1887, III [14]: p. 91). The task of science (2001/1887, III [112]: p. 113) is to produce generalized descriptions of experience: description “is what distinguishes us from older stages of knowledge and science. We are better at describing—we explain just as little as all our predecessors.”

Nietzsche’s *Genealogy of Morality* (2014/1887) predicted that science would overtake religion in prescribing the nature and meaning of life. The results would be catastrophic. Religions regarded life as simply a test for worthiness to enter into an afterlife in the presence of a divine creator or into reincarnation in higher states. The sciences promised new eternal truths that could guarantee the preservation and growth of life here on earth.

Against late nineteenth century’s scientism, Nietzsche argued that all knowledge is based on limited perspectives that cannot deliver final truths. All claims to knowledge are based on assumptions that cannot themselves be given a foundation: “there is no ‘presuppositionless’ knowledge….: a philosophy, a ‘faith’ always has to be there first, for knowledge to win from it a direction, a meaning, a limit, a method, a right to exist” (2014/1887, III [24]: p. 112). Nietzsche insists that our “faith in science” is grounded on a “metaphysical faith” (ibid.). Here Nietzsche anticipates twentieth century claims that knowledge emerges from ‘confabulation.’ As Wilson (2014: p. 157) puts it, ‘Our minds consist of storytelling.’ Both Nietzsche and Wilson agree that the ‘stories’ of science have no claim to final truth, as historians of science have suggested (Kuhn 2012/1963; Cartwright 1983).

Nietzsche could not have anticipated Einstein’s replacement of Newtonian gravitation, but his epistemology readily accommodates that scientific revolution (Clark 1990; Babich 1994; Hussain 2004). Nietzsche feared that the growing European conviction that science could deliver final truth would lead to nihilism. Large populations would come to realize that neither science nor religion can disclose the meaning of life. Nietzsche anticipated the twentieth century as one of wars grounded in rampant nationalism, anti-Semitism and unimaginable technology. He predicted the advent of air travel as an instrument of warfare and globalization (1996/1878, I [477]: p. 176).

Part III of the *Genealogy of Morals* sounds a general warning against science. Nietzsche (2014/1887, III [25]: p. 113) held that only philosophy has the capacity to deliver values: “Science is not nearly independent…, in every respect it first needs a value-ideal, value-creating power, in whose service it can believe in itself,—science itself never creates values.”

Nietzsche even anticipated philosophy’s response to the overwhelming power of science and technology in the twentieth century. Philosophers forsook their primordial mission of telling us how to live and became wardens guarding science from any metaphysical incursions: “Recently they have begun to take pleasure in maintaining that they are actually only the border guards and watchmen of the learned disciplines” (ibid.). Nietzsche calls this “swarm” of border guard philosophers “ridiculous.” But he asks “to what extent are they also harmful? To answer briefly: to the extent that they make philosophy into something ridiculous” (ibid. 250). Indeed, other writings (1995/1874, III [247]: p. 216) express Nietzsche’s fears about the failed future of philosophy: “university philosophy has fallen into universal disrepute and suspicion. … [Y]oung people at our academies will soon be able to get along without philosophy as it is taught at the universities, just as already today men outside the academies are getting along without it.”

Throughout his middle and late periods, Nietzsche argues that philosophy must reclaim its original role of creating value. Neither religion nor science can take up that task. Though strongly under the influence of science in his middle period, he insists that science cannot “build anew the laws of life and of behavior—for this task our sciences of physiology, medicine, sociology … are not yet sure enough of themselves; and only from them can we take the foundation stones for new ideals (if not the new ideals themselves)” (2011/1888, V [453]: p. 212).

Like John Stuart Mill (2014/1859), Nietzsche sets philosophy the task of testing philosophical ground values through experimentation across ranges of cultures. It would be ideal to formulate values grounded in the predictive and controlling force of science. However, that task is premature. We live in an “interregnum” in which “it is best … to found little experimental nations. We are experiments: let us also want to be such!” (ibid.).

And yet in his later period Nietzsche claims that philosophy must rely on science in order both to create new values and to rank values by a hierarchy. In the *Genealogy of Morality* he speaks of the “reserved and suspicious relationship between philosophy, physiology and medicine” (2014/1887, I [17]: p. 34). The philosopher’s task is to convert this antagonism into the “most cordial and fruitful exchange” (ibid.). Philosophy must rely on the sciences in order to make its abstractions from scientific generalizations to ethical prescriptions. Nietzsche goes so far as to say that “All sciences must, from now on, prepare the way for the future work of the philosopher: this work being understood to mean that the philosopher has to solve the problem of values and that he has to decide on the rank order of values” (ibid.).

Science serves as a kind of preparatory course into philosophy by examining history and ethnology through the lenses of physiology, psychology and medical science in general. Such studies will consider the conditions for the “longest possible life-span of a race,” “the improvement of its abilities to adapt to a particular climate,” or “to maintaining the greatest number [of individuals]” (ibid.). The conclusions of these studies cannot yield value prescriptions, inasmuch as the philosopher must decide how these different outcomes are to be ranked. He cites as an example of conflicting values the “good of the majority” versus the “good of the minority.” He berates the “naivety of English biologists” for pronouncing the former as the higher value without having subjected that value to philosophical reflection (ibid). Nietzsche’s contention dating from the unpublished “The Greek State” (1871/1872) up to the *Twilight of the Idols* (1888) is that the good of the minority far outranks that of the majority in a communal pursuit of the will to power as the will to life (Young 2011).

In that early essay he claims that slavery is indispensable to societies dedicated to growth and flourishing of life, rather than its mere preservation. In his late notebooks, he envisioned a thousands of years global European (not German) Reich, the end result of total wars of the twentieth century that he predicted:

It has become possible for international dynasties to emerge which would set themselves the task of rearing a master race, the future ‘masters of the earth’ – a new tremendous aristocracy built upon the harshest self-legislation, in which the will of philosophical men of violence and artist tyrants is made to last for thousands of years: a higher species of men who, thanks to the superiority of the willing, knowing, wealth and influence would make use of democratic Europe as their most tractable and flexible tool to take the destinies of the earth in hand, to sculpt at ‘man’ himself as artists. In short, the time is coming when we will learn to think differently about politics (2003/1885–1888, 2 [57]: p. 71).

In the 2nd edition of the *Gay Science*, Nietzsche (2002/1887, V [377]: p. 241) speaks of the need for a “new slavery—for every strengthening and enhancement of the human type also involves a new kind of enslavement—doesn’t it?”

Nietzsche’s dismissal of compassion and empathy as primordial ethical values justified his rejection of feminism, socialism and democracy. He castigated Darwin’s claim that survival through natural selection is the engine of life. Far more important than survival is growth, the expansion of life, the will to power (2001/1887, V [347]: p. 208). Nietzsche’s vision of social Darwinism minus Herbert Spencer’s claim for compassion justifies the European conquest of the earth.

Walter Kaufman (2013/1950) was right to isolate and conserve what is valuable in Nietzsche’s research. But Nietzsche produced this collection of horrors. Is Nietzsche’s method flawed in itself? Or did the nineteenth century perspectives that drove his use of the method generate these untenable results?

An argument is made in what follows that both are true. Nietzsche’s consilience of philosophy and science tends in the right direction. But it does not go far enough. Wilson’s amplification of Nietzsche’s method corrects Nietzsche’s ill-advised values. But Wilson’s error is to displace philosophy in favor of science.

Science indeed is the platform from which philosophical reflection on value springs. To “become who we are,” in Nietzsche’s (2001/1887, IV [335]: p. 189; see Ridley 2016, pp. 131–133) felicitous phrase, we must “become the best students and discoverers of everything lawful and necessary in the world: we must become physicists in order to be creators in this sense…. So, long live physics!” Nietzsche anticipates a consilience of philosophy and science.

The essay’s next section will examine his conviction that only philosophy can create and rank-order value. Philosophy’s deliberations are to be judged by a ground-value: their ability to promote the growth of life. The status of Nietzsche’s ground-value is questionable. Leiter (2000) argues that Nietzsche does not accord such a value a ‘privileged’ status. [See also Hussain (2004), Clark and Dudrick (2004), Tanesini (2015) for expositions of the metaphysical and semantic status of Nietzsche’s value claims.) Rather he is writing for an audience sympathetic to his views.

The *Gay Science* (2001/1887, I [1]: p. 27) presents Nietzsche’s rationale for his philosophical deliberations on value: Humans dedicate themselves to a “single task, each and every one of them: to do what benefits the preservation of the human race. Not from a feeling of love for the race, but simply because within them nothing is older, stronger, more inexorable and invincible than this instinct—because this instinct constitutes the essence of our species and herd.” Ethical systems, whether springing from mythology, religion, science or philosophy “promote the life of the species by promoting the faith in life” (ibid. p. 28). The drive for life “erupts from time to time as reason and passion of mind” that “tries with all its might to make us forget that fundamentally it is drive, instinct, stupidity, lack of reasons” (ibid.). Our deepest instincts cannot themselves be given reasons: “Life ought to be loved because - ! Man ought to advance himself and his neighbor, because - !” (ibid.). With Darwin, Nietzsche agrees that the preservation of life is a fundamental drive (Richardson 2004). Against Darwin, however, Nietzsche argues that growth through the destruction and creation of values is the only viable instrument of life’s preservation (Johnson 2010). Nietzschean growth trumps Darwinian preservation—in the service of preservation.

## Does Philosophy have a Proprietary Right to the Creation and Rank-Ordering of Value?

Does Nietzsche have an argument for his claim that philosophy alone has the power to create and rank-order values? History furnishes a mixed answer to this question. Philosophers like Plato set reason as the most important commanding value. Ancient Greek hedonists and nineteenth century utilitarians set pleasure above all other values. Kant, Hegel and Marx found freedom to be the foundational value. But religious figures like the Hindu sages, the Buddha, Christ and Muhammad created and rank-ordered their own values.

Do philosophers have some sort of special powers over value that religious figures lack? Capitalizing on Socrates’ example, philosophers may argue that their value inventions and rankings may be justified by the rigorous exercise of reasoned argument. Reason, pleasure and freedom seem to be values indispensable for preservation and growth. But how are they to be rank-ordered through argument? Nietzsche (2001/1887, III [121]: p. 121) claims that the deepest values cannot be touched by reason, that life itself is no argument: “We have arranged for ourselves a world in which we are able to live—by positing bodies, lines, planes, causes and effects, motion and rest, form and content; without these articles of faith no one could endure living! But that does not prove them. Life is not an argument; the conditions of life might include error” (see also 2001/1887, I [1]: pp. 27–29). Must reason be more important than pleasure—or are they to be co-valent? Must we exercise reason and pleasure in the service of freedom? What form of argument could make that conclusive demonstration?

Darwin himself made claims for large-scale, tightly bonded communities. Darwin’s first principle is that we are able to survive and flourish only through the power of our groups. Other things being equal, the larger the groups, the better their chances of surviving and flourishing. The second principle is that the more tightly bonded together the members of our groups are, the better the groups’ chances: “… a tribe including many members who … were always ready to give aid to each other and to sacrifice themselves for the common good, would be victorious over most other tribes; and this would be natural selection” (Darwin, *Descent of Man*, cited in Wilson 2016: p. 210). In the extreme case, members will sacrifice their lives for the sake of their groups. Compulsory conscription for military service in wartime encodes that truth into law.

Creating value cannot be the exclusive province of the philosopher. In his last two essays, the American pragmatist Richard Rorty (2009a, b) asserts that Shakespeare and Cervantes have done more to advance human progress than the combined works of all the philosophers. Artists create values that displace old values, as a cursory glance at the history of art will show.

And a primary task of scientists, engineers and technologists is to create the values that direct their research and implementation programs. Those values are deeply controversial among their own communities, as the historic conflicts over quantum mechanics and contemporary disputes over string theories suggest. Researchers in mathematics create new values that transform the discipline through non-Euclidean geometries and Kurt Gödel’s claims of inconsistency and incompleteness in complex formal systems. Quantum logic and quantum super-computing bring new values to the field of logic. And Noam Chomsky’s claims for a universal grammar introduce a new value to the field of grammar.

Religious figures, artists of all stamps, scientists, engineers, technologists, historians, mathematicians, logicians, grammarians—all create and rank-order values. Historians, psychologists, sociologists and anthropologists attempt to trace the genealogies of these value-systems. How can Nietzsche claim that the creation, rank-ordering and genealogies of values constitute the special province of the philosopher?

His only hope is to justify his model through the methodological principles of Plato and Aristotle. Like Plato, Nietzsche pursues a value that gives birth to all other values. Plato’s transcendent value is the ‘good.’ Inspired by Darwin’s ‘naturalization’ of life, Nietzsche dismisses Plato’s ground value as a pure abstraction. He substitutes the “will to power,” defined as “the will to life” (2014/1887, II [12]: p. 52). Philosophers are those who create values that promote the growth of life, thereby ensuring its preservation. Artists, religious figures, scientists, may inspire the philosopher’s creations, but it is the philosopher’s task alone to rank-order the values that are grounded in the “will to life.”

Like Aristotle, Nietzsche believes that the philosopher’s right to create and rank-order values comes from what Aristotle calls a generalized knowledge of all things (*Metaphysics*, 982a9–10). Plato calls that same kind of knowledge “synoptic vision” (*Republic*, 534b). Like his predecessors, Nietzsche imagined that he as a thinker possessed sufficient ‘wisdom’ or ‘total knowledge’ to justify his judgments about value. However, the *Gay Science* (2001/1887, V [351]: p. 210) cautions against taking any value including Nietzsche’s own as a final value: “it was modesty that in Greece coined the word ‘philosopher’ and left the extraordinary insolence of calling oneself wise to the actors of the spirit—the modesty of such monsters of pride and conceit as Pythagoras, as Plato –.”

Given the early twentieth century explosion of scientific knowledge, Nietzsche was perhaps the last thinker who could try to make a provisional claim to wisdom. The *Gay Science* (2001/1887 V [381]: p. 246) expresses his fear that “we philosophers are all in a bad position regarding knowledge these days; science is growing, and the most scholarly of us are close to discovering that they know too little.”

In the *Genealogy of Morality*, Nietzsche (2014/1887, I [17]: p. 34) states that philosophy should act as an “advocate” for selected values issuing from scientific research. The corrective measure for Nietzsche’s method for creating and rank-ordering values is to take his admonitions about the consilience of philosophy and science more seriously than he did. Against Nietzsche’s dismissal of compassion and empathy, Edward O. Wilson argues that self-sacrifice for the group is a genetic disposition under the right circumstances. Wilson claims that this disposition rather than kin selection explains altruistic acts. Wilson imagines that his scientific research answers the philosophical question about human values.

## Wilson’s Attempt to Bridge the Fact/Value Gap Through Science

In contrast to Nietzsche, Edward O. Wilson (2014) in the *Meaning of Human Existence* argues that science can now conclusively answer philosophy’s deeply controversial questions about human values. For Wilson, both philosophy and science are based on stories that we construct with our imaginative powers. He speaks of the “human necessity for confabulation. Our minds consist of storytelling” (ibid. p. 167). The purpose of the stories is to reduce the “flood of real-world information” that flows into our brain to actionable patterns that allow us to predict and control our experience. The information presented by the senses “far exceeds what the brain can process.” Wilson would agree with Aldous Huxley that the brain is a ‘reducing valve.’ We create competing stories, comparing them with past experience, to determine how to project ourselves into the future. The stories “are weighed against one another by the suppressing or intensifying effect imposed by aroused emotional centers” (ibid.).

Wilson agrees with Nietzsche on a fundamental point. We live by the stories we tell ourselves: “Conscious mental life is built entirely from confabulation. It is a constant review of stories experienced in the past and competing stories invented for the future. By necessity most conform to the present real world as best it can be processed by our rather paltry senses” (ibid. pp. 167–168).

Scientific certainty appears to replace the massive uncertainty of philosophical speculation. However, scientific descriptions can only be pragmatically confirmed. Newton’s ‘universal laws’ were ‘true’ only for their limited range of action. Einstein replaced Newton’s more speculative assumptions (infinite space, rectilinear motion, action at a distance, Euclidean geometry) with his own (finite but unbounded space, curvilinear motion, distortion of space by mass, Riemannian geometry) to achieve better conformity with the scope and simplification rules of rationality.

Newton’s laws of gravitation stood unassailable for centuries despite their inability to cover anomalies like Mercury’s orbit around the sun. Advanced astronomical measuring techniques showed that Newton’s laws can’t describe the motion of fast ‘bodies’ such as photons or massive objects such as the sun. Newton’s ‘universal’ generalizations are still useful for space exploration but his philosophical assumptions belong to the graveyard of “failed models” (Wilson’s term for the history of philosophy, 1998: pp. 160–161).

Science conveys the illusion of delivering certainty against the failure of philosophy to escape from uncertainty. What props up that illusion? Science delivers the capacity to predict and control experience through its generalized descriptions. However, we can achieve goals by acting on misinformation. Imagine thinking that malaria (from mal and aire) is caused by poisonous air generated at night by standing water in swamps. Sealing bedroom windows and doors against the night air might help to stop malaria. But draining the swamp water would be an even more effective remedy. Acting on false beliefs about malaria could eliminate the disease, absent any understanding of the night-borne anopheles mosquito as the transmission vector.

If as Wilson claims ‘our minds consist of storytelling,’ are there any kinds of stories that are proscribed? Wilson notes the thousands of creation myths generated by religion. He explains the origin of these myths as a “Darwinian device for survival” (1998: p. 8). Their primary function is to serve as the bonding power of religious groups. If both science and religion are “confabulations,” can they ever be reconciled? Wilson’s answer is straightforward: “They cannot be reconciled. Their opposition defines the difference between science and religion, between trust in empiricism and belief in the supernatural” (ibid.).

If religion is impotent to explain the meaning of human existence, may philosophy serve? Wilson (1998: p. 9) says “we look in vain to philosophy for the answer to the great riddle.” He imagines that “pure philosophy long ago abandoned the foundation questions about human existence. The query itself is a reputation killer. It has become a Gorgon for philosophers, upon whose visage even the best thinkers fear to gaze” (ibid.). The rationale for contemporary philosophers’ desertion of their primordial mission? Philosophers have tried to solve the riddle of existence using “failed models of the mind” that produce the “wreckage of theories of consciousness” (ibid.). When logical positivism declined in the mid-twentieth century, philosophers in a grand diaspora “emigrated into the more tractable disciplines not yet colonized by science—intellectual history, semantics, logic, foundational mathematics, ethics, theology, and, most lucratively, problems of personal life adjustment” (ibid: pp. 9–10).

Wilson claims that only science has the capacity to solve the riddle of human existence. He (2012: p. 10) elaborates his scientific solution in *The Social Conquest of the Earth* through a broad examination of the “other social conquerors of Earth, the highly social ants, bees, wasps, and termites.” He recognizes that his research program will be greeted with some apprehension: “I realize I can be easily misinterpreted by putting insects next to people. Apes are bad enough, you might say, but insects?” (ibid.).

What the “social conquerors of the Earth” have in common is a genetic disposition to sacrifice individual or close-kin interest for the sake of group interest. He came to this research program by rejecting his earlier attempts to explain altruistic behavior through Hamilton’s theory of inclusive fitness.

Hamilton’s theory holds that natural selection targets individuals. It served as Wilson’s controlling assumption for the research project that came to be called sociobiology (Wilson 2000/1975, 1978; Lumsden and Wilson 1983). The opposing theory holds that natural selection targets groups. Wilson’s new theory calls for multilevel selection or a contest between individual and group fitness. Wilson believes that group selection has prevailed in human history because of humanity’s ‘eusocial’ nature. As with two species of African mole rats, fourteen species of insects, and three shrimp species, the preponderant human inclination is to sacrifice individual for group interest (2014: p. 111).

Wilson’s change in position was so repugnant to his fellow researchers committed to inclusive fitness theory that 137 of them signed a rejection of his new theory in *Nature* (ibid: p. 73). Richard Dawkins, whom Wilson calls a “science journalist” (ibid: p. 70), urged readers of Wilson’s *Social Conquest of the Earth* “to cast the entire book away, ‘with great force,’ no less” (ibid: p. 73).

For Wilson, the meaning of human existence is altruistic or eusocial behavior. Selfish behavior promotes individual fitness but destroys the group. Altruistic behavior promotes group interest. The contest between the two behaviors is hardwired into our genes and fueled by our emotions. Wilson goes so far as to equate individual selection with sin and group selection with virtue: “The result is the internal conflict of conscience that afflicts all but psychopaths, estimated fortunately to make up only 1–4% of the population” (ibid: p. 179). The result of this contest? “All normal humans are both ignoble and noble, often in close alternation, sometimes simultaneously” (ibid: p. 180).

Wilson’s paradigmatic revolution, so stoutly resisted by his fellow researchers in biology, illustrates the inseparable nature of philosophy and science. First, through his application of the rules for rational theory selection, he has replaced a prevailing theory through a new theory, although both theories ground themselves in principles of natural selection.

Second, Wilson imagines that his new theory has the power to explain traditional contests between good and evil. In his view, selfish and altruistic behavior correlate with sin and virtue. Here he offers a scientific explanation for judgments in the field of ethics.

Third, he generalizes from a claim about the eusocial nature of humanity to a universal declaration about the meaning of human existence. After condemning philosophical attempts to define human nature, he furnishes his own definition which must be philosophical by reason of its extreme generality. He claims empirical support for his extreme generalization by reason of the interplay between individual and group selection. Wilson claims that eusociality is a primary cause of humanity’s conquest of the earth. Wilson simply cannot help philosophizing: The “involuted exercises and professional timidity’ of Western philosophy ‘have left modern culture bankrupt of meaning” (1998: p. 269).

Wilson proposes a course correction for the liberal arts. They must address “the fundamental questions of human existence head on, without embarrassment or fear, taking them from the top down in easily understood language, and progressively rearranging them into domains of inquiry that unite the best of science and the humanities at each level of organization in turn” (1998: p. 269). Wilson (2017: p. 8) in his most recent work imagines that his consilience of the two fields in a new Enlightenment “can at last solve the great questions of philosophy.” One can only ask, what have the liberal arts been engaged in since time immemorial, if not “addressing the fundamental questions of human existence head on”? Wilson’s spectacular leap from a controversial hypothesis about human eusociality to the meaning of human existence dismisses the limits of knowledge. One hundred and thirty-seven biologists reject his hypothesis. His platform for a secular humanism is problematic at best. The fates of millennia of assumptions about the meaning of existence show that answers to this question must be provisional and subjected to vast amounts of experimentation, as both Nietzsche and Mill recommend.

Can contemporary science add a new and perhaps consensual value that augments or supplants these directions for humanity’s growth? On the one hand, Nietzsche (2001/1887, V [357]: p. 219) insists that centuries must pass before the question about the meaning of existence “can ever be heard completely and in its full depth.” On the other hand, he (2002/1886, IX [252]: p. 158) claims that new systems of morals and ethics arise out of great dangers: “Again danger is there, the mother of morals, great danger…. The greater the danger is, the greater is the need to reach agreement quickly and easily about what must be done.” For the first time in human history, we have now created the gravest of dangers: the human-caused sixth mass extinction (Kolbert 2014). We may hope that the consilience of the sciences and philosophy has the power to arrest our course.

Philosophy is neither a science nor one of the humanities. Rather it is a bridge, fulcrum or balance point between the two. In proposing bold new ways to live, philosophy tilts in the direction of imagination and artistry. The philosophies that appear to have controlled the greatest number of humans over the past five thousand years must be seen as utterly preposterous by those not raised within their geographical precincts. They are perfect expressions of the art of imagination: live a life wholly dedicated to pleasure, reason, love, freedom, survival, or a balance of these life objectives. Nietzsche (2001/1887, I [1]: pp. 28–29) speaks of these classical human values as “so foolish and contrary to nature that humanity would have perished from every one had it gained power over humanity…. … There is no denying that in the long run each … was vanquished by laughter, reason and nature….” Nietzsche cautions us against philosophy’s temptation to exaggeration but virtually all of these human values are far from being ‘vanquished’ in the present moment.

From the other side, philosophy suggests promising directions to turn the preposterous into common sense. Philosophy tilts in the direction of science, engineering and technology. To cite examples: The earth moves, contrary to what ‘common sense’ appears to tell us. Euclidean geometry is insufficient to help describe the universe’s fabric. Mass and velocity bend space and time. Converting mass into energy (*E *= *mc*^2^) can produce unimaginable chaos.

Nietzsche (2001/1887, I [7]: pp. 34–35) anticipates Wilson’s claim that science may overshadow philosophy in creating and rank-ordering values. Scientists will study “why does the sun of one fundamental moral judgment and primary value-standard shine here—and another one there….” The larger project “would be to determine the erroneousness of … the whole essence of moral judgments to date” (see Collins 2000). The crux is whether science can replace philosophy in setting goals for action. Such a question, Nietzsche says, can only be resolved by experiments requiring the work of centuries. Such experimentation would “eclipse all the great projects and sacrifices of history to date. So far science has not yet built its cyclops-buildings; but the time for that will come too.”

The next section examines three contemporary models for the consilience of science and philosophy. In the *Gay Science* (2001/1887, III [112]: p. 113), Nietzsche speaks of the false dichotomy of cause and effect: “there is probably never such a duality; in truth a continuum faces us, from which we isolate a few pieces….” Thinkers like Wilson impose the same duality on philosophy and science, but they too form an unbreakable bond.

## Three Contemporary Models of the Consilience of Philosophy and Science

Like Plato, Aristotle and Nietzsche, Richard Rorty (2016: pp. 59–60), one of Nietzsche’s ablest disciples in the twentieth century, summarizes philosophy’s method as an “attempt at synoptic vision.” Rorty takes his inspiration from Wilfrid Sellars’ (1962: p. 1) proposal that philosophy consider “how things in the broadest possible sense of the term hang together.” Philosophy for Rorty “must take the form of a supernarrative” that synthesizes the histories of science, politics, poetry, theology, architecture as well as history and philosophy themselves (ibid.).

Rebuking scientists like Hawking, Dawkins, and deGrasse Tyson, Rorty reminds us that “philosophy…always buries its undertakers.” Why? Because “the attempt to achieve a synoptic vision of human achievement will never cease.” The idea that a single academic discipline like philosophy can accomplish that task, however, “may be on its way out” (ibid: p. 61). The twentieth century’s explosion of knowledge dictates that only a consilience of philosophy and science can achieve such a vision. Historically, because of the limits of knowledge, a single person might engage all the disciplines necessary to dictate a path for life, in the tradition of the Buddha or Aristotle, Christ or Kant. Because of the explosion of knowledge in both the humanities and STEM fields, only teams of researchers can pragmatically discharge philosophy’s functions.

Three contemporary philosophers model this consilience. They rebut Nietzsche’s claim that only philosophy is competent to create and assess values, and Wilson’s claim that science is now capable of declaring the meaning of human existence—with empirical verification.

Evan Thompson’s *Life of Mind* (2010) ranges across neuroscience, cognitive science, psychology and biology using a phenomenological method. His grasp of these disciplines expresses a model of consilience of philosophy and science. His research shows the hollowness of Wilson’s claim that philosophy relies on failed models of the mind or brain.

Although he was a former critic of Edmund Husserl, Thompson uses phenomenological techniques to connect life and mind, nature and consciousness. He synthesizes objective descriptions of life with our subjective impressions. He follows a path all the way from neural organization to consciousness and culture. His work is a triumph over the Cartesian legacy of separating mind from life, spirit from matter.

Robert Frodeman and Adam Briggle’s  (2016) *Socrates Tenured: The Institutions of twenty*-*first Century Philosophy* promotes a vision of a ‘field philosophy’ that unites philosophy not simply to other disciplines but to the wider world of unsolved problems in areas of politics, economics and social justice. The authors claim that Anglo-American philosophy labors under the burden of its disciplinary and departmental locations. The extraordinary progress of science in the twentieth century inclined philosophers toward specializations in logic, analytic philosophy and philosophy of language. In the 1980s, groups of philosophers turned to environmental ethics and bioethics to apply their methods to wider problems. In the twenty-first century, those problems become existential in the face of global climate change.

Frodeman and Briggle envision field philosophy as taking philosophers out of their departments to join with non-academic institutions such as government organizations, NGOs, think tanks, businesses, laboratories, communities—in short, a host of organizations that might find the philosopher’s synoptic vision useful for problem-solving. Philosophers might also join other departments with emphasis on the sciences, medicine, law and engineering. The field philosopher’s research would depend on the nature of the problems the academic and non-academic fields address. The authors’ proposal primarily targets Anglo-American philosophers. Paul-Michel Foucault’s research on genealogies of various kinds of human experience and the Frankfort school’s dialogical methods may serve as models for the field philosopher.

One question the authors might have explored more thoroughly is the competence philosophers may bring to the solution of academic problems outside of philosophy and non-academic problems in the wider world. Philosophers from Plato and Aristotle through René Descartes, Immanuel Kant and Georg Wilhelm Friedrich Hegel offered syntheses of the compendium of knowledge of their times. They stand as historic models of philosophy as synoptic vision that encompasses both global questions about primordial values and detailed prescriptions for the pursuit of research in all the academic disciplines. Our contemporary problem is that the compendium of knowledge has expanded far beyond the grasp of a single person.

Dale Jamieson’s *Reason in a Dark Time: Why the Struggle against Climate Change Failed*—*and What It means for Our Future* (2014), is a model for using consilience to address an existential problem. A distinguished philosopher at New York University, Jamieson presents a history of climate change science, as well as reviews of the political and economic obstacles to addressing the problem. He offers a ‘common sense morality’ as the ethical platform of his observations.

Jamieson specifies a number of reasons for climate change policy failure, ranging from failures to understand climate science, connecting science to policy, profiteering from greenhouse gas emissions, and the absence of a unified international political institution. The problem requires global collective action. The United Nations simply cannot command that power.

Evolutionary biology poses quite another problem. Jamieson (2014: p. 4) claims that “evolution built us to respond to rapid movements of middle-sized objects” rather than slow increases in atmospheric gases. Contemporary ethics and economics are not geared to assess the long-range consequences of climate change: “The idea that turning up my thermostat in New York can contribute to affecting people living in Malaysia in a thousand years is virtually beyond comprehension to most of us” (2014: p. 102). Jamieson’s prognostications about a thousand-year future for life on earth will seem overly optimistic if methane bubbling out of the Arctic slopes and sea combines with CO_2_ to form a nearly impermeable greenhouse blanket. The earth has experienced that scenario in previous mass extinctions. Jamieson (2014: p. 1) insists that climate change will cause no more deaths than the twentieth century catastrophes if we are “lucky.”

Jamieson’s research does not offer a specific model for arresting climate change. The recommendations he makes in the final chapter are well-publicized: carbon capture and sink, carbon and methane pricing, government mandated renewable energy, increased government and private sector energy research. Nevertheless, his command of the disciplines required to propose and assess climate change policy is a powerful illustration of Frodeman and Briggles’ field philosophy. As his title suggests, Jamieson’s research is a reflection on the existential problem of climate change. He does not offer a call to action based on a plausible model. What follows offers a field philosophy model for solving quite another existential problem.

## Conclusion: An African Model for Humanity’s Future

Migration from the world’s most war-torn and impoverished countries drives a resurgence of nationalism in the developed nations. Around a billion humans experience food insecurity. Their children undergo the life-long consequences of stunting as their brains fail to develop. Some three billion have no access to toilets. Their children suffer from water-borne disease because of their immature immune systems. The New York Times reports five million deaths in the Eastern Congo since 1998 because of conflicts over coltan and tantalum, metals critical for cell phones and jet turbine fan blades.

Can a consilient philosophy begin to confront these kinds of existential challenges? An African model developed by Professor Godfrey Nzamujo, a Dominican priest who embodies the practice of ‘field philosophy’ in the spirit of Frodeman and Briggle, is now considered. Committed to helping the rural poor in Africa, Nzamujo prepared for the task by taking degrees in philosophy, mathematics, systems engineering, electrical engineering, computer science, economics and microbiology. While field philosophy requires researchers from numerous disciplines, Nzamujo brought a number of those disciplines together in his own person. Not believing that training for his work in development requires PhDs, he says he entered his PhD programs because he needed the discipline.

Nzamujo located his field philosophy model on the outskirts of Porto Novo, Benin in 1988. The model takes the form of an agro- or eco-village that synthesizes the cultural heritages of Africa with selective resources from the global store of knowledge. The village is called the Songhai Center in honor of the West African Songhai Empire.

The Songhai Center has seven objectives:

- Produce food in sufficient quantity and quality to promote health;
- Enhance the environment (soil life, food web, soil structure, and the like);
- Provide a framework for a new settlement model: Agro-Hoods or Green Rural Cities;
- Build sustainability and biodiversity;
- Provide feed stock for renewable energy;
- Create employment, particularly for the young and women (Nzamujo 2018).

While the village is rooted in the physical and cultural soil of Africa, it relies on advanced technology. Using techniques for water conservation, soil regeneration, irrigation and cultivation, the village produces abundant enriched food for its residents and for export to other areas. One example of Nzamujo’s agro-technology is using infra-red (IR) and ultra-violet (UV) resistant sheets to kill weeds without pesticides. He perforates the same sheets in cultivation rows to ensure optimum soil temperature and hydration. A primary source of fertilizer is effluent from the tilapia and catfish aquaculture ponds. He uses water hyacinth plants for stage one recycling of that water. The plants are then absorbed in a biogas digester. With bacterial soil nutrients and carbon root attachment nodules, Nzamujo is able to bring a pineapple crop to maturity in 1 year rather than the 2–3 year cycle of conventional crops. The Center includes a foundry for fabricating agricultural implements and a state of the art facility for Information Communication Technology (ICT).

Nzamujo believes that rural Africans must take responsibility for their own grounds for survival and flourishing. These include a supportive community, aesthetic clothing and housing, clean air and water, medical care and education. The center is not only autochthonous but autonomous, that is, responsible for its own sustainability. To that end, Nzamujo views the Center as a “knowledge enterprise.” The key is primary and advanced schooling for the residents that includes agro-ecology, business practices, ICT, and an ethics of responsibility for community members as well as the wider communities in which the Center is located.

Especially important for the economic health of the model is the Songhai Regional Center Marketing and Sales Platform. While the original Songhai Center in Porto Novo aims at autonomy, it is guided by a philosophy of interdependence not only with all other Centers, but also the wider communities in which the Centers are embedded. They serve surrounding communities with agricultural extension services and market information. With an emphasis on entrepreneurship the Service Center has six objectives:

- Deploy its information network to relate critical market information to participants;
- Promote sub-sectors and commodities chains, new product line and market niches;
- Undertake group purchases, transport and storage facilities to reduce costs;
- Build a virtual stock system within the network to ensure delivery of orders in the network;
- Engage local banks and innovative financial institutions to reduce transaction costs and increase network capital;
- Foster collaboration between network members and agro-industrial and agri-business groups in their regions of operation (Nzamujo 2018).

The Songhai Center model has spread from Porto Novo to several other locations in Benin and Togo. With extraordinary rapidity, it has also blossomed in more than fifteen other countries in Africa including Burkina Faso, Chad, Democratic Republic of the Congo, Equatorial Guinea, the Gambia, Ghana, Guinea, Liberia, Malawi, Nigeria, Republic of the Congo, and Uganda.

The ethics that ground the Songhai model spring from Nzamujo’s commitment as a Dominican priest to be of service to under-resourced communities. Deeply versed in Thomas Aquinas’ (himself a Dominican) transformation of Aristotelian ethics, Nzamujo takes Teilhard de Chardin’s concept of human evolution from the “biosphere” to the “noosphere” as his model. The knowledge embodied in the ‘noosphere’ has reached a point where humanity may now transform itself from *Homo sapiens* to *Homo empathicus*. Conceiving of the Songhai Centers as “knowledge enterprises,” Nzamujo believes that this evolutionary progression is contingent on advancing knowledge in philosophy, the STEM disciplines and the humanities. The educational platform for the Centers is based on the principle of field philosophy, applying theoretical knowledge from academic research programs to practical applications with the promise to solve existential problems.

Nzamujo’s model offers the promise of sustainable food production for the accelerating earth’s population. With its ICT connectivity to the global store of knowledge, the Songhai eco-village offers a life of excellence to the poorest rural areas in the Global South. Capitalizing on the principle that “waste is wealth,” the village combines solar power with biomass and biogas energy sources to move toward a zero carbon footprint (McDonough and Braungart 2002).

The model is not without its challenges (Vodouhe and Zoundji 2013). The original Songhai Center requires energy from the diesel-powered Porto Novo grid. The advanced agricultural technology, the laboratories, production and business centers, and especially the ICT facilities require intensive start-up capital. Nzamujo has been resourceful in raising funds for the complete array of centers from the United Nations, governments, NGOs and private foundations. In 2015, the UN Food and Agriculture Organization awarded its 70th anniversary commemorative medal to Nzamujo in recognition of his achievements in promoting sustainable development, prosperity and wellbeing. He presents a model of consilient philosophy in action with the potential to confront the existential crisis of poverty in the Global South.

Does the Songhai Center model have the potential to spread out of Africa to the poorest areas of Asia, South America and Oceania? The critical question is whether humanity will adopt an ethics that guarantees human survival. Nietzsche (2011/1888, II [108]: p. 75) said that there can be no common ethics for humanity because no one can agree on a universal ethical principle: “Only if humanity had a universally recognized goal could one propound ‘such and such should be done’: for the time being, there is no such goal.” Humanity’s goals, continues Nietzsche, must be “in keeping with our own discretion.” And humanity can “give itself a moral law—likewise, in keeping with its own discretion” (ibid.).

Are we on the threshold of that new moral law? Nietzsche (2011/1888, V [460]: p. 234) speaks of “taking advantage of one’s dangerous hours.” He imagined that his own times would not permit such an imperative: “Nowadays we all live, comparatively speaking, in much too great a safety to become real knowers of humanity” (ibid.). We are now on the threshold of what he might have anticipated: “Know or be destroyed!” Our “truths” begin to “slice themselves into our flesh with knives” and we can begin to abandon our “secret disdain for them…” (ibid.).

Perhaps Nietzsche did not imagine that humanity would develop the technological power to create the sixth mass extinction, although he questions humanity’s capacity to guarantee a future. He speaks (2001/1887, V [362]: p. 227) of our having “entered the classic age of war, of sophisticated yet popular war on the largest scale (in terms of weapons, talents, discipline)….” Human madness is such a powerful “counter-drive” to rationality “that it is basically with little confidence that one may speak of the future of humanity” (ibid: II [76]: p. 77).

Nietzsche certainly did not imagine that twenty-first century technology would have the power to reduce resource scarcity, displace human labor with artificial intelligence and robotics, and enable virtually universal higher education with increasing amounts of leisure coupled with universal broadband access and information communication technology. Massive open on-line courses offered by research universities like Harvard and Stanford, Oxford and Cambridge are a halting first step toward this goal (Bowen 2013).

The question of moment is whether sufficient numbers of humans have the will to pass life on to our children’s children. Nietzsche’s claim that philosophers are “guarantors of the future” exaggerates the power of a philosophy that does not intimately link itself to the STEM disciplines and humanities. Against both Nietzsche and Wilson, only teams of researchers embedded in and informed by their larger communities are qualified to make decisions about which new values are to be implemented through the services of science, engineering and technology. Such teams must embrace the needs and visions of the communities for whom they are responsible. The creation of new values, even new values that serve to ground wholesale value systems, is not a task exclusive to philosophers.

Philosophy’s forte is Plato’s “synoptic vision” that brings these teams and these communities together (*Republic* 534b). With Plato, Nietzsche (2001/1887, V [343]: p. 199) portrays the philosopher as a “lookout at the top of the mountain, posted between today and tomorrow.” He speaks of philosophers as “firstlings and premature births of the next century, to whom the shadows that must soon envelop Europe really should have become apparent by now….” (ibid.) As Nietzsche (2014/1887, II 12: p. 52) foresaw, philosophy buoyed up by that vision must act as an advocate for ever-changing values that are ultimately grounded in the growth of life that guarantees its preservation. Adaptation to evolution’s selective pressures follows only upon “the spontaneous, aggressive, expansive, re-interpreting, re-directing and formative forces” under the guidance of humanity’s creation and destruction of values.

The impetus for such a consilience must spring from philosophy. From Plato and Aristotle to Nietzsche and Rorty, philosophy’s method can never change. However, technology’s growing power forces a change in philosophy’s mission. Given the existential threats of global climate change and weapons of mass destruction, we must acknowledge Nietzsche’s pessimism, as quoted above: “it is basically with little confidence that one may speak of the future of humanity” (ibid: II [76]: p. 77). Against that pessimism, philosophy must stand as “guarantor of the future” (2014/1887, III [14]: p. 91).

## Footnotes

1 Citations for Nietzsche’s texts include in sequence publication date of translation, original date of publication, book or part number, section number, and page number in the translated edition.

2 The term *value* is used here in the conventional sense of beliefs or ideals about what is good or bad, desirable or undesirable. Values are the emotive and reasoned drives of human experience and behavior.
